# *In Vitro* Ability of a Novel Nanohydroxyapatite Oral Rinse to Occlude Dentine Tubules

**DOI:** 10.1155/2015/153284

**Published:** 2015-06-03

**Authors:** Robert G. Hill, Xiaohui Chen, David G. Gillam

**Affiliations:** ^1^Dental Physical Sciences, Barts and The London School of Medicine and Dentistry, Queen Mary University of London (QMUL), London E1 4NS, UK; ^2^School of Dentistry, The University of Manchester, Manchester M13 9PL, UK; ^3^Centre for Adult Oral Health, Barts and The London School of Medicine and Dentistry, Queen Mary University of London (QMUL), London E1 2AD, UK

## Abstract

*Objectives*. The aim of the study was to investigate the ability of a novel nanohydroxyapatite (nHA) desensitizing oral rinse to occlude dentine tubules compared to selected commercially available desensitizing oral rinses. *Methods*. 25 caries-free extracted molars were sectioned into 1 mm thick dentine discs. The dentine discs (*n* = 25) were etched with 6% citric acid for 2 minutes and rinsed with distilled water, prior to a 30-second application of test and control oral rinses. Evaluation was by (1) Scanning Electron Microscopy (SEM) of the dentine surface and (2) fluid flow measurements through a dentine disc. *Results*. Most of the oral rinses failed to adequately cover the dentine surface apart from the nHa oral rinse. However the hydroxyapatite, 1.4% potassium oxalate, and arginine/PVM/MA copolymer oral rinses, appeared to be relatively more effective than the nHA test and negative control rinses (potassium nitrate) in relation to a reduction in fluid flow measurements. *Conclusions*. Although the novel nHA oral rinse demonstrated the ability to occlude the dentine tubules and reduce the fluid flow measurements, some of the other oral rinses appeared to demonstrate a statistically significant reduction in fluid flow through the dentine disc, in particular the arginine/PVM/MA copolymer oral rinse.

## 1. Introduction

Dentine hypersensitivity (DH) is a clinical problem that may impact on the quality of life of individuals who may experience discomfort when eating and drinking hot and cold food and drink during their day to day activities [1]. Currently there is no recognized ideal desensitizing product (over-the-counter [toothpaste or mouthwash] or dentist [professionally] applied) that provides both fast acting and long lasting protection against the pain associated with DH [2]. This concern has subsequently led to the development of novel substances or reformulation of existing technologies for example, bioactive glasses (Novamin) and hydroxyapatite/nanohydroxyapatite/nanocarbonate apatite crystal toothpastes (HAP) [3–7], Colgate Sensitive Pro-Relief toothpastes and oral rinses [8–11], and a 1.4% potassium oxalate oral rinse (LISTERINE Advanced Defence Sensitive) [12–15]. Currently toothpastes, gels, and oral rinses are designed to reduce or relieve pain arising from DH based on either their (1) tubular occluding or (2) nerve desensitization properties. Recently a number of novel nanohydroxyapatite toothpastes and oral rinses (nHAs) have been developed for home use (over-the-counter [OTC]) and these products may be an attractive alternative to the traditional desensitizing toothpastes and oral rinses for treating DH [16, 17]. The aim of the present study therefore was to investigate the ability of a novel nanohydroxyapatite desensitizing oral rinse to occlude dentine tubules and reduce fluid flow in comparison with other selected commercially available desensitizing oral rinses.

## 2. Materials and Methods

25 caries-free extracted maxillary and mandibular molars were obtained from the tooth bank at the Royal London Dental Hospital, London, UK. The* in vitro* occlusion of the dentine tubules was investigated using the dentine disc model [18]. The teeth were sectioned mesiodistally into discs approximately 1 mm thick using an internal edge annular diamond blade (Microslice annular blade, Ultratec, USA) mounted on the Microslice 2 saw (Malvern Instruments Ltd., UK) and halved (test and control sections). The dentine discs (*n* = 25) were etched with 6% citric acid for 2 minutes and rinsed with distilled water for 30 seconds, prior to the application of the test and control oral rinses. Each disc was divided into a control and test section by masking half the disk prior to application of the selected oral rinses for 30 seconds (*n* = 5 samples per group). Evaluation of the tubule occluding ability of the test and control oral rinses was by (1) Scanning Electron Microscopy (SEM) of the dentine surface and (2) measurement of the fluid flow (hydraulic conductance) through the dentine disc before and after 30 seconds of rinsing of the test and control oral rinses. One disc from each of the test and control oral rinses was used in the hydraulic conduction aspect of the study; each disc was subjected to 5 evaluations.

### 2.1. Materials

Five oral rinses, namely, two Hydroxyapatite based nHA (UltraDEX Recalcifying & Whitening, Periproducts Ltd., UK) and zinc substituted HA (BioRepairMicroRepair BioRepair/ACDOCO) oral rinses, potassium oxalate (1.4%) (LISTERINE Advanced Defence Sensitive, Johnson and Johnson Inc., New Brunswick, New Jersey, USA), arginine and PVM/MA copolymer oral rinse (Colgate Sensitive Pro-Relief, Colgate-Palmolive, UK), and a negative control oral rinse containing potassium nitrate (Sensodyne Pronamel Daily Mouthwash, GSK Consumer HealthCare, Weybridge, UK), were evaluated. The nHA powder used for the novel hydroxyapatite oral rinse (UltraDEX Recalcifying & Whitening) was also supplied separately by Periproducts Ltd., UK. A commercial high purity sintered hydroxyapatite powder (Captal R) was obtained from Plasma Biotal Ltd. (Matlock, Derbyshire, UK) as a reference. The remaining test and negative control formulations were obtained commercially.

Tubule occlusion was assessed as described above using Scanning Electron Microscopy (SEM) and by measuring the fluid flow (hydraulic conductance [Lp]) through the dentine discs using a modified Pashley hydraulic conductance model [19, 20].

#### 2.1.1. Evaluation of the Dentine Specimens by Tubule Occlusion (Scanning Electron Microscopy [SEM])

The methodology used for preparation follow those described by Gillam et al. [21]. Half the dentine disc section was masked and the remaining half treated with the oral rinse/mouthwash for 30 seconds and then rinsed in distilled water for a further 30 s (dilution factor 1 : 2). The samples were then dried and gold coated prior to SEM examination.

#### 2.1.2. Effectiveness of Tubule Occlusion Using Hydraulic Conductance (Fluid Flow) Measurements

The use of hydraulic conductance evaluation in order to assess the dentine fluid flow has been established by Pashley and coworkers [19, 20] and is a useful* in vitro* method recognized by the American Dental Association (ADA) to evaluate both desensitizing toothpastes and oral rinses that work via a dentine occlusion mechanism. Fluid flow can be measured using a Pashley cell hydrodynamic flow device [19, 20]. The tests were performed on 1 mm thick dentine discs cut from the mid coronal section of human molars. The dentine discs (*n* = 5; one per test and control group) were then etched using 6% citric acid for 2 minutes to remove the smear layer and open the tubules. The disc was then mounted in the Pashley cell and the fluid flow measured over time.

### 2.2. Analysis of the Fluid Flow Measurements

The differences between the flow rates between the test and control oral rinses were analysed by Student *t*-test. Mean and Standard deviations of the test and control oral rinses were also assessed.

## 3. Results

### 3.1. Scanning Electron Microscopy (SEM) of the Effects of the Test and Control Oral Rinses on Tubule Occlusion

**Figure 1:**
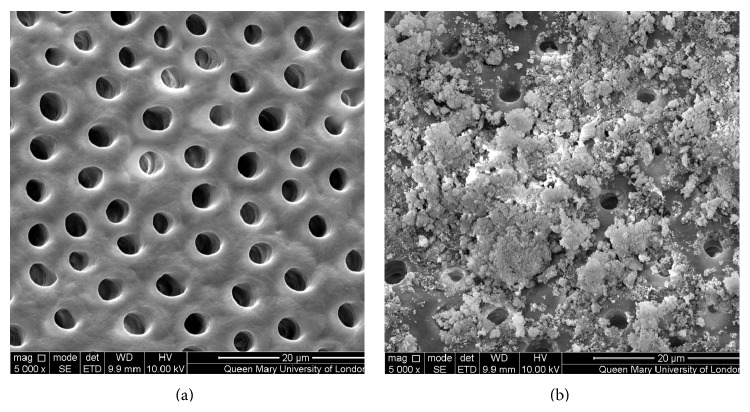
SEM images of dentine tubules treated with nHA oral rinse (UltraDEX HA Recalcifying oral rinse), before treatment (a) and after treatment (b).

**Figure 2:**
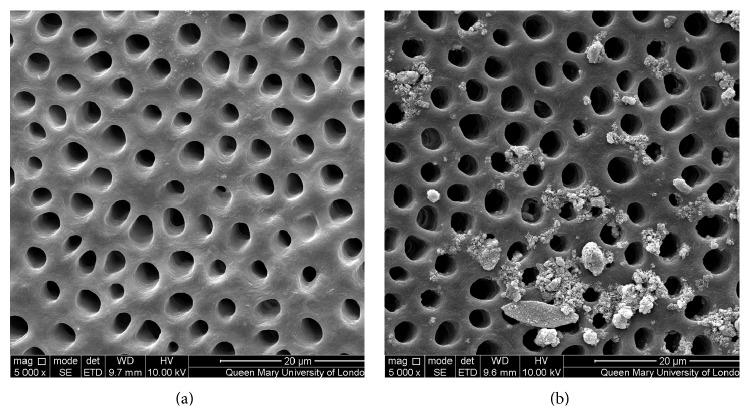
SEM images of dentine tubules treated with a zinc substituted HA oral rinse (Biorepair MicroRepair Mouthwash), before treatment (a) and after treatment (b).

**Figure 3:**
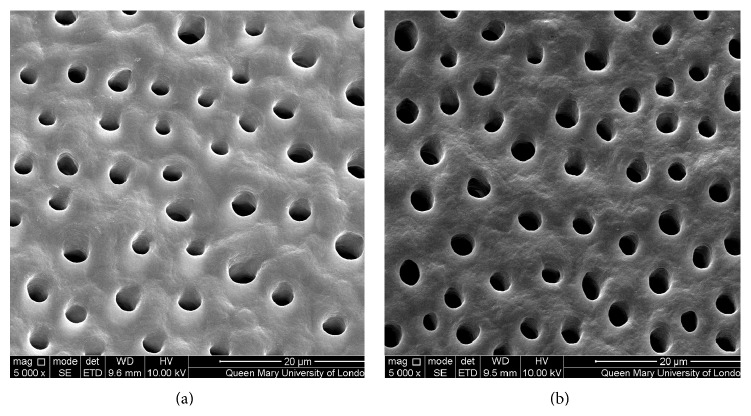
SEM images of dentine tubules treated with arginine and PVM/MA copolymer oral rinse (Colgate Sensitive Pro-Relief Mouthwash), before treatment (a) and after treatment (b).

**Figure 4 fig4:**
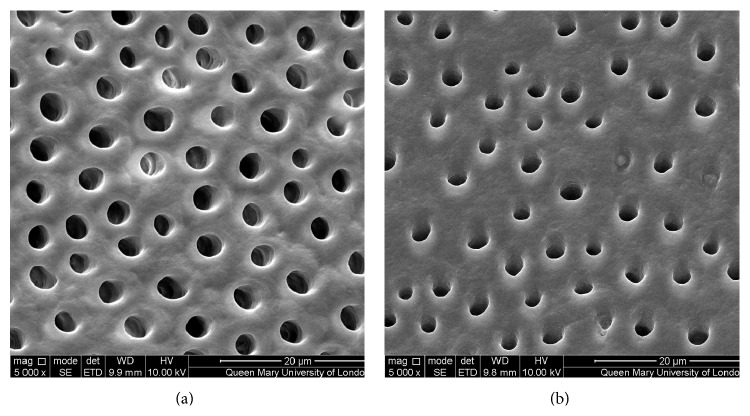
SEM images of dentine tubules treated with a 1.4% potassium oxalate oral rinse (LISTERINE Advanced Defence Sensitive Mouthwash), before treatment (a) and after treatment (b).

**Figure 5:**
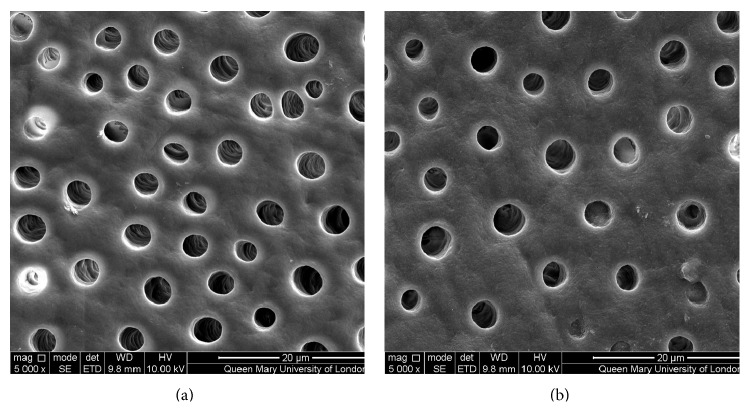
SEM images of dentine tubules treated with a potassium nitrate oral rinse (Sensodyne Pronamel Daily Mouthwash), before treatment (a) and after treatment (b).

Figures 1–5 show the SEMs of the dentine discs before and after treatment with the test and control oral rinses. When treated with the nHA oral rinse for 30 seconds a number of the dentine tubules were occluded by the HA particles as observed in Figure 1. The HA particles appear to cover the dentine surface of the dentine disc as well as penetrate into the dentine tubules. The zinc substituted HA oral rinse (BioRepair MicroRepair Mouthwash) (Figure 2) however provided a less dense particle coverage and occlusion of the dentine tubules compared to the nHA oral rinse (Figure 1); this may have been as a consequence of the much lower HA concentration in this oral rinse. In comparison, the other oral rinse formulations do not appear to demonstrate any clear evidence of occlusion of the dentine surface compared with the etched control (before treatment) (Figures 3 [Fig fig4]–5).

### 3.2. Hydraulic Conductance Fluid Flow Measurements

The fluid flow reduction (FFR) results for the desensitizing oral rinses were recorded in Table 1. Student *t*-tests (*p* < 0.05) were used to analyze the data from the results.

## 4. Discussion

There have been concerns previously expressed in the literature that the current strategies for treating DH may not provide a lasting solution to the problem of both tooth surface loss and pain associated with DH [2]. According to Gillam [15] a number of novel substances or reformulation of previously described technologies have been reintroduced into the consumer market, for example, bioactive glasses (Novamin) and hydroxyapatite/nanohydroxyapatite/nanocarbonate apatite crystal toothpastes (HAP) [3–7], Colgate ProArgin toothpastes and oral rinses [8–11], and a 1.4% potassium oxalate mouth rinse (LISTERINE Advanced Defence Sensitive) [13, 14]. More recently a novel nanohydroxyapatite formulation (UltraDEX Recalcifying & Whitening toothpaste and oral rinse) has been developed [16, 17]. According to Hill et al. [16, 17] the advantages of using hydroxyapatite in a toothpaste and oral rinse formulation would be that as a natural component of tooth and bone, the HA component has the ability to be incorporated into the tooth structure [16]. For the purposes of evaluating this new desensitizing formulation, the Investigators used a dentine disc methodology to determine the effectiveness of a number of commercially available oral rinses in reducing fluid flow through tubular occlusion. The zinc substituted HA was included to compare the effectiveness of a commercially available oral rinse: both the arginine and PVM/MA copolymer and 1.4% potassium oxalate oral rinses have been reported to be effective in reducing DH and were used as positive controls [10, 11, 13, 14] and the potassium nitrate oral rinse was included as a negative control as its mode of action is generally considered to be by nerve desensitization rather than by tubular occlusion. In order to test the potential ability of a desensitizing product prior to clinical evaluation,* in vitro* and/or* in situ* studies are often conducted in order to determine a possible mode of action of the product. One of the problems however when conducting* in vitro* evaluation outside the oral environment is that it is very difficult to completely mimic the dynamics and interaction of saliva, and so forth. For example, most* in vitro* studies will evaluate the surface deposit of a particular formulation using Tris buffer, artificial saliva, and so forth, when brushing or rinsing with a test or control formulation, and will report on the ability of these products to occlude the dentine tubule. However it may be possible that this observation alone may be ineffective in identifying any potential of effectiveness in the oral environment when a particular formulation interacts with the saliva over time. Furthermore reporting on the surface precipitation or deposit alone without evaluating the hydraulic conductance measurements following the application of a particular desensitizing product may also be misleading. According to the principles underpinning the hydrodynamic theory fluid flow through dentine is inversely proportional to 1/radius^4^ and therefore relatively small reductions in the functional radius of the tubule diameter will have a significant effect on fluid flow. In the present study it was observed that not all of the test and control formulations covered the dentine surface (Figures 1–5) although it was evident from the fluid flow measurements (Table 1) that a degree of tubular occlusion must have occurred occurred below the dentine surface in order to have an effect on this change. One of the criticisms of the present study, however, would be that no investigation was undertaken to determine whether there was a degree of subsurface occlusion that would account for this observation. This particular phenomenon has been reported in the published literature for potassium oxalate previously used for in-office applications to treat DH [22–24].

The results from the SEM analysis of the test and control oral rinses provided a wide variation in the manner and extent of the surface precipitate (Figures 1–5). For example, the novel nHA and zinc substituted HA demonstrated varying degrees of surface coverage whereas potassium nitrate formulation failed to demonstrate any surface deposition which may be explained as indicated above that the recognized mode of action is through reducing nerve desensitization rather than by tubule occlusion. Both the arginine and PVM/MA copolymer and 1.4% potassium oxalate oral rinses also failed to show any surface deposit although as previously described it is more likely that there was subsurface precipitation within the dentine tubules which may explain why these two formulations demonstrated reductions in the fluid flow measurements (Table 1).

All the oral rinses investigated in the present study to some degree resulted in a fluid flow reduction (Table 1). For example, the arginine and PVM/MA copolymer oral rinse (66.9%) was the most effective in reducing FFR values compared to the 1.4% potassium oxalate (41.7%), nHa (40.3%)/zinc substituted HA (48.7%), respectively. It was apparent from these results that the arginine and PVM/MA copolymer oral rinse was statistically significant compared to the new nHA (*p* = 0.0002) and the 1.4% potassium oxalate (*p* = 0.0036) oral rinses, respectively, although there were no statistically significant differences between the new nHA/zinc substituted HA 1.4% potassium oxalate oral rinses. The negative control formulation (potassium nitrate) resulted in the least reduction in FFR values (24%) and all the other oral rinses demonstrated statistically significant differences in FFR in relation to the negative control (arginine and PVM/MA copolymer oral rinse (*p* = 0.0000), 1.4% potassium oxalate (0.0051), zinc substituted HA (*p* = 0.0087), and nHA (*p* = 0.0098)), respectively. This observation is perhaps not surprising given that its primary mode of action does not involve a tubule occlusion mechanism. There does, however appear to be a limited correlation between the observed tubule occlusion observed by SEM and the measured FFR values and this is as a result of the SEM methodology used in the present study being more appropriate for the physical deposition of the particles rather than the differences in the mode of action for the other tested oral rinses. Furthermore the variations within the dentine disc particularly with the differences in the FFR values (Table 1) are primarily due to the anatomical variations (in terms of tubule size and tubule orientation) within the dentine disc itself which may complicate obtaining an ideal surface to evaluate these products. In the present study one dentine disc was evaluated for each oral rinse (*n* = 5) when assessing the FFR values in order to limit the variation in testing between different dentine discs. This may have accounted for the differences between the published values in the literature.

For example, Sharma et al. [14] investigated the composition used in the 1.4% potassium oxalate formulation and reported that the oral rinse demonstrated a 55% reduction in FFR values following three treatment applications and an approximately 100% reduction after 12 treatment applications. The 55% reduction compares reasonably with the 42% FFR reduction reported for one treatment in the present study. Mello et al. [11] investigated the hydraulic conductance of a prototype oral rinse similar to the arginine and PVM/MA copolymer oral rinse formulation. These investigators used a very different procedure from that used in the present study and included two treatments of 10 minutes which appeared excessive for an oral rinse application and reported a 59% reduction in FFR values. This percentage reduction is reasonably comparable to the 67% reduction demonstrated for the arginine and PVM/MA copolymer oral rinse in the present study following one treatment application.

Eliades et al. [13] used 12 treatment applications when investigating the tubule occluding properties of an arginine and PVM/MA copolymer oral rinse using back scattered SEM; there was however no significant evidence of tubule occlusion reported either on the dentine surface or in the subsurface of the dentine section. These investigators also investigated the 1.4% potassium oxalate formulation and in contrast to Sharma et al. [14] study there was little evidence of tubule occlusion at the surface, but there was, however, evidence of crystals in the tubules beneath the dentine surface as well as a degree of tubule occlusion which is consistent with the evidence reported in the literature [22–24].

A criticism of both the existing studies and the present study was the use of sections of mid coronal dentine for the studies of tubule occlusion and hydraulic conductance. For example, clinically DH is diagnosed on the buccal (facial) surfaces of the cervical region of the tooth where the dentine tubule size is generally considered to be smaller in diameter and has a different orientation from the tubules in the mid coronal region of dentine. Furthermore acid etching of the dentine surface prior to treatment not only removes the smear layer and opens up the tubules but also makes the openings to the tubules more funnel shaped in nature that may aid particles and liquids entering the tubules. Root dentine taken from the cervical region of the tooth would therefore be a more appropriate model than mid coronal sections; however, as indicated above there is greater variability in terms of tubule orientation of the root dentine compared to mid coronal dentine. A further problem as observed in the studies using the dentine disc model was the difficulty in reproducing the hydraulic conductance measurements. One possible method of reducing this variation in scatter would be to measure the flow rate prior to and after the application of the oral rinse using the same disc as indicated above, although the variation in scatter may be still evident as reported in the present study.

## 5. Conclusions

Although the new nanohydroxyapatite (nHA) demonstrated the ability to both occlude the dentine tubules and reduce the fluid flow values, nevertheless some of the other oral rinses appeared to demonstrate a statistically significant reduction in fluid flow through the dentine disc, in particular the arginine and PVM/MA copolymer oral rinse compared to the other test and control rinses.

## Figures and Tables

**Table 1:** Fluid flow reduction measurements for the test and control oral rinses (*n* = 5).

Oral rinse	Mean FFR (%)	SD (%)
UltraDEX Recalcifying & Whitening	40.3	9.6
BioRepair MicroRepair	48.7	15.9
LISTERINE Advanced Defence Sensitive	41.7	14.2
Colgate Sensitive Pro-Relief	66.9	6.6
Sensodyne Pronamel Daily Mouthwash	24	9.3
